# Real-world treatment patterns and survival outcomes in patients with locally advanced squamous cell carcinoma of the head and neck in Germany using claims data

**DOI:** 10.3389/fonc.2025.1547311

**Published:** 2025-06-05

**Authors:** Kathrin Hering, Thomas Kuhnt, Nils Kossack, Lena M. Richter, Michael Schultze, Ulrike Osowski, Luisa Henkel, Ann-Christin Gaupel, Marie-Noelle Solbes, Bernard Zolyniak, Nancy Schoenherr

**Affiliations:** ^1^ Department of Imaging and Radiation Medicine, Clinic of Radiooncology, University of Leipzig, Leipzig, Germany; ^2^ WIG2 Institute for Health Economics and Health System Research, Leipzig, Germany; ^3^ ZEG – Berlin Center for Epidemiology and Health Research GmbH, Berlin, Germany; ^4^ Merck Healthcare Germany GmbH, an affiliate of Merck KGaA, Weiterstadt, Germany; ^5^ Merck Healthcare KGaA, Darmstadt, Germany

**Keywords:** squamous cell carcinoma, head and neck cancer, locally advanced, real-world evidence, overall survival, database analysis, resection, chemotherapy with radiotherapy

## Abstract

**Introduction:**

Standard-of-care treatment for locally advanced squamous cell carcinoma of the head and neck (LA SCCHN) is surgery with consolidation chemoradiotherapy (CRT) or definitive CRT. There is a paucity of real-world evidence regarding current treatment patterns and downstream outcomes for LA SCCHN in German clinical practice.

**Methods:**

This study was a non-interventional, observational, retrospective cohort study of newly diagnosed patients with LA SCCHN using routinely collected claims data from a health insurance claims database in Germany (2016–2021). Claim records were used to describe the cohort, including incidence, characteristics, treatment patterns, and survival. As permitted by the data, descriptive analyses were stratified by index treatment (surgical resection or definitive non-surgical treatment), tumor site (oral cavity, oropharynx, hypopharynx, or larynx), and sex. The study was descriptive in nature; as such, no statistical comparisons were made.

**Results:**

The LA SCCHN cohort comprised 1,010 patients (827 male and 183 female patients), of whom 39.8% (402/1,010) received surgical resection and 60.2% (608/1,010) received definitive non-surgical treatment as part of index treatment. Patients with surgical resection as part of index treatment were characterized by a younger mean age and lower comorbidity indices. After index treatment, three-quarters (74.8%) of the study population received no subsequent SCCHN treatment. Index treatment was similar for male and female patients. The rate of surgical resection and definitive non-surgical treatment was similar in patients with oral cavity cancer [50.6% (128/253) and 49.4% (125/253), respectively]; all other tumor sites were treated more frequently (>60%) with definitive non-surgical treatment. The 5-year probability of survival for the overall population was 48.5% (95% CI: 44.4%–53.1%). Survival probabilities varied across tumor sites and by index treatment.

**Conclusion:**

Despite index treatment being broadly aligned to guideline recommendations, most patients did not receive a subsequent line of treatment and almost half of patients had died within 5 years. This highlights the urgent unmet need for improved treatment options for LA SCCHN.

## 1 Introduction

Head and neck cancer is a broad term for malignant tumors that occur in the upper aerodigestive tract (1). The majority of head and neck cancers are squamous cell carcinomas (SCCHN) that arise from epithelial cells in the oral cavity, oropharynx, hypopharynx, and larynx (1, 2). Five-year survival for each is estimated at 48%, 41%, 25%, and 61%, respectively (3).

SCCHN is the seventh most common cancer worldwide, with rising incidence attributed to an increase in oropharyngeal cancer linked to human papillomavirus (HPV) infection (2, 4, 5). However, tobacco use and alcohol consumption remain the major risk factors, responsible for 75%–85% of cases (2). Globally, head and neck cancer is associated with a 3:1 male:female ratio (6). According to the Global Cancer Observatory, in 2022, there were almost 16,000 new cases of head and neck cancer in Germany (7); however, there is a paucity of SCCHN-specific epidemiology data.

Stage at diagnosis, tumor site, histology, and certain patient characteristics all inform the prognosis and treatment of SCCHN (2, 8), leading to a highly heterogeneous patient population. Of note, HPV-related oropharyngeal cancer is known to be associated with a substantially better outcome than other SCCHN types, independent of the treatment received. This has led to a relatively recent distinction of HPV-related oropharyngeal cancer as a separate tumor entity; however, application of the new staging system has presented challenges in practice (9–11).

Unfortunately, approximately two-thirds of patients with SCCHN are diagnosed at the locally advanced (LA) stage (12, 13). The aim of treatment for patients with LA SCCHN is to achieve cure at the lowest risk of morbidity (2, 14). Standard of care at this stage, as recommended by European and German clinical guidelines, is surgical resection plus adjuvant radiotherapy or chemoradiotherapy (CRT), or definitive non-surgical treatment (i.e., definitive CRT) (2, 15–17). The latter approach is reserved for patients who cannot or choose not to undergo surgery, for example, due to an inoperable tumor, preservation of function, or comorbidities, and has been the mainstay approach for unresected SCCHN for more than 30 years (2, 18–20). Over half of patients with LA SCCHN develop local recurrence and/or distant metastases within 2 years of completing treatment (12, 21, 22); treatment at this stage is largely palliative (23–25).

Heterogeneity in the presentation of patients with respect to demographics, particularly sex, and tumor site inherently results in variation in the approach to, and therefore outcomes of, treatment in German clinical practice. This study sought to understand Germany-specific LA SCCHN epidemiology and the types, sequences, and survival outcomes of treatments received in real-world practice using data from a public health insurance claims database.

## 2 Methods

### 2.1 Study period and data collection

This non-interventional, observational, retrospective, longitudinal cohort study described epidemiology, patient characteristics, treatment patterns, and clinical outcomes among patients with newly diagnosed LA SCCHN in German clinical practice between 1 January 2016 and 31 December 2021.

The Wissenschaftliches Institut für Gesundheitsökonomie und Gesundheitssystemforschung (WIG2) Institute Research Database was used for this study; this is an anonymized healthcare claims database comprising data for approximately 4.5 million persons insured by a German statutory health insurance (SHI) provider. It includes the demographics of insured persons in addition to healthcare service use. At the time of study conduct, the database contained data from 1 January 2014 to 31 December 2021 with a low attrition rate (26).

The index date was defined as the first date of a head and neck cancer diagnosis (≥1 inpatient diagnosis or ≥2 confirmed outpatient diagnoses) within the index period (1 January 2016 and 31 December 2020). A 12-month baseline period prior to diagnosis was used to capture patient baseline characteristics. A post-index period of at least 12 months (except in case of discontinuation or death) was used to evaluate treatment patterns and outcomes.

### 2.2 Inclusion and exclusion criteria

Since databases used for insurance claims purposes do not routinely capture tumor stage at diagnosis, comprehensive inclusion and exclusion criteria were designed to identify incident patients with LA SCCHN among all registered patients with head and neck cancer based on treatments received and sequences thereof. This was informed by German and European guidelines (2, 15–17, 27) and clinical expert opinion. Full inclusion and exclusion criteria can be found in **Supplementary Material Section 1**.

#### 2.2.1 Inclusion criteria

The study included patients with an initial diagnosis of head and neck cancer defined as ≥1 International Classification of Diseases 10th edition German Modification (ICD-10-GM) (28) diagnostic code of inpatient diagnosis or ≥2 confirmed outpatient diagnoses of head and neck cancer within 365 days. Patients were aged ≥18 years at first diagnosis (index date) and had ≥24 months of continuous enrolment prior to the index date (to ensure at least 24 months without prior diagnosis of head and neck cancer).

#### 2.2.2 Exclusion criteria

The study excluded patients with cancers other than those of the head and neck, ambiguous tumor site diagnoses, or ICD-10-GM codes for metastatic cancer (28). The German SHI outpatient billing system works in quarters; therefore, patients without any treatment for SCCHN in the quarter after first diagnosis of LA SCCHN or the subsequent quarter were excluded. Patients who had received initial treatment indicating early-stage cancer (i.e., surgical resection only or chemotherapy only) or metastatic disease were excluded. Patients participating in a clinical trial were also excluded.

### 2.3 Study outcomes and cohorts

#### 2.3.1 Study outcomes

##### 2.3.1.1 Epidemiology

The incidence of LA SCCHN in the database was extrapolated to provide an estimate of the incidence of LA SCCHN in the German population insured by an SHI. Baseline demographic, clinical, and disease characteristics of the database cohort were described.

##### 2.3.1.2 Treatment types and pathways

Description of treatment types and pathways included characterization of the index treatment regimen received and up to two subsequent treatment regimens post-index treatment. Systemic treatments received were characterized, where feasible; however, inpatient treatments are commonly billed as part of a lump sum, precluding delineation when using insurance claims data.

A treatment algorithm was constructed to define index and subsequent treatment strategies. Patients were considered to be treated if treatment for SCCHN was initiated within 6 months of the index date. The first treatment received after the index date was considered index treatment. Index treatment was defined as any treatment modality (surgery, radiotherapy, or systemic treatment) administered within 90 days of the start of the first treatment. The end date of index treatment was defined as the latest date of the last treatment received. In patients receiving systemic treatment as part of index treatment, additional agents initiated within 8 days of the first systemic treatment were considered part of the same systemic treatment regimen. Treatment was considered as concurrent CRT if systemic treatment and radiotherapy were given within 14 days of each other. A switch of systemic treatment led to advancing the treatment line, i.e., from index treatment to subsequent lines, with the exception of a switch from cisplatin to carboplatin. The end of index treatment was defined by either a switch or a discontinuation (>60-day gap between prescriptions).

Treatment pathways from index treatment through subsequent regimens were captured as Sankey plots.

##### 2.3.1.3 Survival

Survival outcomes as 1-, 3-, and 5-year survival probabilities were described.

#### 2.3.2 Cohorts

##### 2.3.2.1 Epidemiology

Baseline demographic, clinical, and disease characteristics were assessed for the overall study population, as well as by index treatment received.

##### 2.3.2.2 Treatment types and patterns

Index treatment was split into two cohorts defined by primary treatment received: surgical resection or definitive non-surgical treatment, which were further stratified by sex and primary tumor site.

Treatment pathways were described for the overall study population and by index treatment.

##### 2.3.2.3 Survival

Survival outcomes were assessed for the overall study population, as well as by index treatment and primary tumor site.

### 2.4 Data analysis

Descriptive analyses were performed to characterize cohort characteristics and treatments. Discrete variables were summarized using frequencies and proportions, and continuous variables were summarized using means and standard deviations.

Survival time-to-event analyses were conducted using Kaplan–Meier methods. Death was considered a competing risk to a next treatment. Patients who changed insurer or did not have a date of death documented within the study were censored at the end of the observation period.

Because of the expected heterogeneity in the population and treatment approaches, the study was designed to be descriptive in nature with no statistical comparisons between or within cohorts.

## 3 Results

### 3.1 Study population

**Figure 1:**
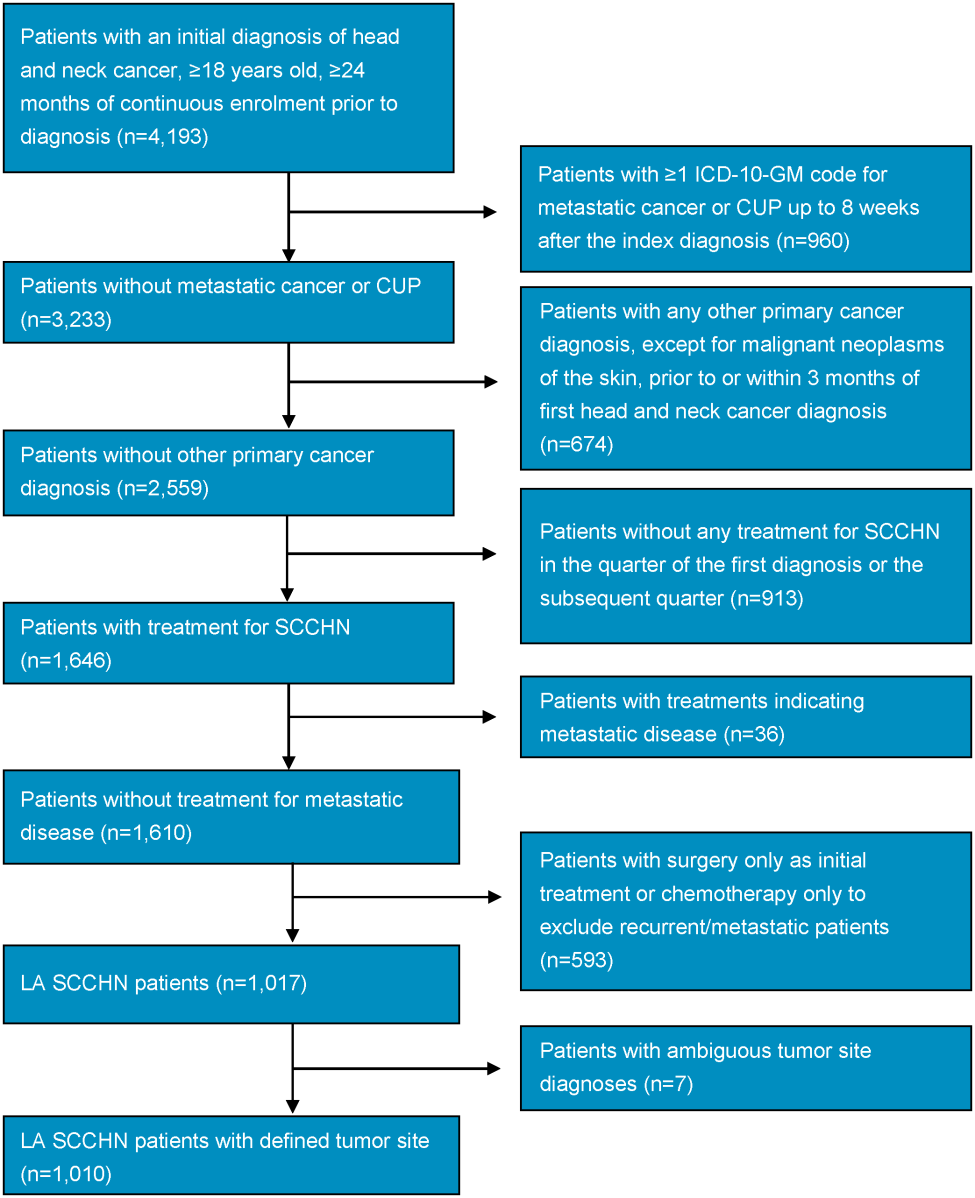
Study population attrition flowchart. CUP, cancer of unknown primary; ICD-10-GM, International Statistical Classification of Diseases and Related Health Problems, 10th revision, German Modification; LA, locally advanced; SCCHN, squamous cell carcinoma of the head and neck.

**Figure 1** shows the study population attrition. Initially, 4,193 patients with a diagnosis of head and neck cancer were identified in the database during the index period. After the application of inclusion and exclusion criteria, 1,010 patients with LA SCCHN remained. The study comprised 183 female patients, equating to roughly a ~4:1 male:female ratio.

### 3.2 Epidemiology

#### 3.2.1 Incidence

The incidence of LA SCCHN ranged from 7.10 to 9.04 per 100,000 insured in the database per year over the study period. When extrapolated to the SHI population, the incidence per 100,000 SHI-insured adults was 6.66 in the first year (2016) and 7.70 in the final year of the index period (2020), peaking at 8.35 in 2018 (**Supplementary Table S3**).

#### 3.2.2 Baseline characteristics

Demographic, clinical, and disease characteristics at initial diagnosis for the overall study population and stratified by index treatment are described in **Table 1**.

**Table 1:** Baseline demographic, clinical, and disease characteristics by overall study population and index treatment.

Characteristic	Overall study population (*N* = 1,010)	Surgical resection (*N* = 402)	Definitive non-surgical treatment (*N* = 608)
Age (years), mean (SD)	62.5 (9.47)	61.3 (9.44)	63.4 (9.41)
Age categories, *n* (%)			
≤65	647 (64.1)	278 (69.2)	369 (60.7)
>65	363 (35.9)	124 (30.8)	239 (39.3)
≤70	800 (79.2)	334 (83.1)	466 (76.6)
>70	210 (20.8)	68 (16.9)	142 (23.4)
Sex, *n* (%)			
Male	827 (81.9)	331 (82.3)	496 (81.6)
Female	183 (18.1)	71 (17.7)	112 (18.4)
Primary tumor site, *n* (%)			
Oropharynx	389 (38.5)	134 (33.3)	255 (41.9)
Oral cavity	253 (25.0)	128 (31.8)	125 (20.6)
Larynx	238 (23.6)	94 (23.4)	144 (23.7)
Hypopharynx	130 (12.9)	46 (11.4)	84 (13.8)
Comorbidity indices, mean (SD)			
ECI	6.2 (8.38)	5.5 (8.06)	6.7 (8.56)
CCI	2.3 (2.77)	2.1 (2.64)	2.4 (2.84)
Risk factors, *n* (%)			
Tobacco consumption	303 (30.0)	128 (31.8)	175 (28.8)
Alcohol abuse	263 (26.0)	106 (26.4)	157 (25.8)
Most common comorbid conditions at diagnosis or within 2 years prior, *n* (%)			
Diabetes	222 (22.0)	76 (18.9)	146 (24.0)
Mild liver disease	216 (21.4)	87 (21.6)	129 (21.2)
Peripheral vascular disease	199 (19.7)	70 (17.4)	129 (21.2)

CCI, Charlson Comorbidity Index; ECI, Elixhauser Comorbidity Index; SD, standard deviation.

The observed patient population consisted of predominantly male patients (81.9%) with an advanced age (mean, 62.5 years). Oropharyngeal cancer was the most common tumor type (38.5%), followed by oral cavity cancer (25.0%), and laryngeal cancer (23.6%); cancer of the hypopharynx was the least common (12.9%). Tobacco consumption and alcohol abuse were respectively reported for 30.0% and 26.0% of the total population. Of the comorbidities considered as part of this study, the most frequent were diabetes (22.0%), mild liver disease (21.4%), and peripheral vascular disease (19.7%).

Upon holistic consideration of the data, patients who had received surgical resection as part of index treatment were numerically slightly younger (mean, 61.3 versus 63.4 years, respectively) and had lower comorbidity indices [mean Elixhauser Comorbidity Index (ECI): 5.5 versus 6.7, respectively; mean Charlson Comorbidity Index (CCI): 2.1 versus 2.4, respectively] than patients who received definitive non-surgical treatment.

When considering the characteristics of female and male patients with LA SCCHN (**Supplementary Table S4**), the mean age at diagnosis was numerically similar (63.1 versus 62.4 years, respectively); however, fewer female patients presented with alcohol abuse (14.2% versus 28.7%, respectively) and tobacco use (26.8% versus 30.7%, respectively) at baseline. Female patients presented with a higher percentage of oral cavity (31.1% versus 23.7%) and oropharyngeal (43.2% versus 37.5%) cancers, whereas male patients presented with a higher percentage of laryngeal (25.3% versus 15.8%) and hypopharyngeal (13.5% versus 9.8%) cancers.

### 3.3 Treatment types and patterns

In the overall study population, 402 (39.8%) patients received surgical resection and 608 (60.2%) patients received definitive non-surgical treatment as part of index treatment (**Table 1**). There was no numerical difference in the rate of surgical resection and definitive non-surgical treatment between male and female patients (**Supplementary Table S4**).

**Figure 2:**
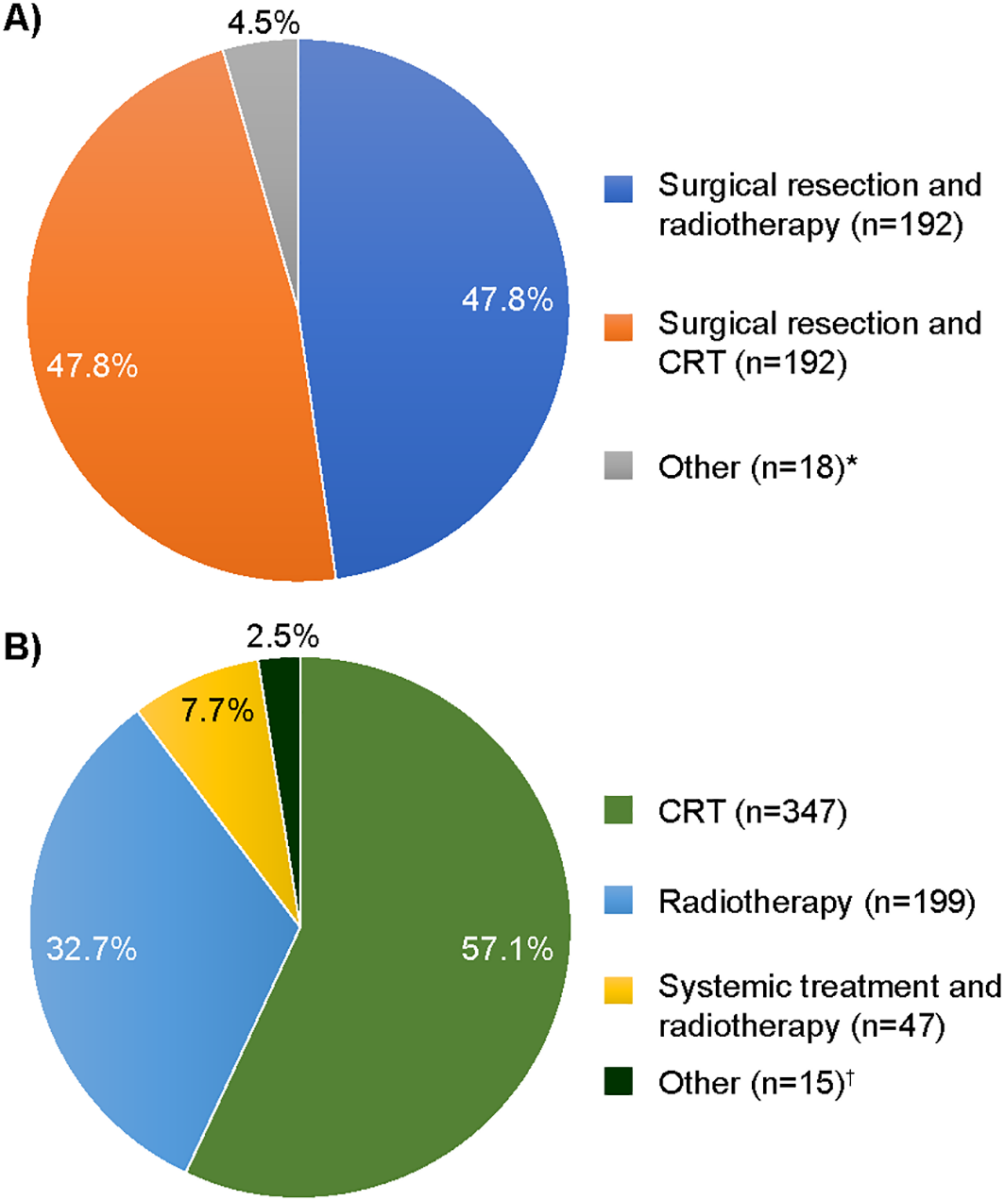
Index treatment regimens received by **(A)** surgical resection [*N* = 402 (% >100 due to rounding)] and **(B)** definitive non-surgical treatment (*N* = 608). CRT, chemoradiotherapy. *”Other” included surgical resection and systemic treatment ± radiotherapy; ^†^”Other” included CRT or radiotherapy and surgery (assumed to be salvage surgery due to sequence of treatments).

The majority [95.5% (384/402)] of patients in the surgical resection group also received adjuvant radiotherapy [47.8% (192/402)] or CRT [47.8% (192/402)] as part of index treatment (**Figure 2**). For patients who did not receive surgical resection, the most common index treatment regimen was definitive CRT [57.1% (347/608)], or definitive radiotherapy [32.7% (199/608)], while other treatment options were less common. In patients for whom systemic treatment was possible to identify (*N* = 246), cisplatin was the most common systemic index treatment [76.4% (*n* = 188)], followed by cetuximab [15.0% (*n* = 37)] (**Table 2**).

**Table 2:** Systemic treatments received as part of index treatment.

Treatment(s)	Patients with identifiable systemic treatment(s)*		
	Overall study population (*N* = 246)	Surgical resection (*N* = 92)	Definitive non- surgical treatment (*N* = 154)
Cisplatin, *n* (%)	188 (76.4)	79 (85.9)	109 (70.8)
Cetuximab, *n* (%)	37 (15.0)	7 (7.6)	30 (19.5)
Cisplatin + other^†^, *n* (%)	12 (4.9)	<5 (N/A^‡^)	9 (5.8)
Other^†^, *n* (%)	9 (3.6)	<5 (N/A^‡^)	6 (3.9)

N/A, not applicable.

*Limitations in the claims database prevent the identification of certain inpatient treatments (see Section 4.1); ^†^“Other” included the following chemotherapy regimens: carboplatin, carboplatin + fluorouracil; carboplatin, paclitaxel; cisplatin, docetaxel; fluorouracil, mitomycin C; ^‡^Some groups could not be reported for data protection purposes (*n* < 5); therefore, % could not be reported.

Index treatment regimens by primary tumor site are shown in **Table 3**. The rate of surgical resection versus definitive non-surgical treatment was similar for patients with oral cavity cancer [50.6% (128/253) versus 49.4% (125/253), respectively], whereas higher rates of definitive non-surgical treatment were seen in patients with tumors originating in the oropharynx [65.6% (255/389) versus 34.4% (134/389)], hypopharynx [64.6% (84/130) versus 35.4% (46/130)], and larynx [60.5% (144/238) versus 39.5% (94/238)]. Of definitive non-surgical treatments for cancers of the oral cavity, oropharynx, and hypopharynx, definitive CRT was the most common treatment strategy, whereas patients with laryngeal cancer commonly received definitive radiotherapy.

**Table 3:** Index treatment regimen by primary tumor site.

Treatment(s)	Oral cavity (*N* = 253)	Oropharynx (*N* = 389)	Hypopharynx (*N* = 130)	Larynx (*N* = 238)
Surgical resection, *N* (%)	128 (50.6)	134 (34.4)	46 (35.4)	94 (39.5)
Surgical resection and adjuvant radiotherapy, *n* (%)	74 (57.8)	55 (41.0)	14 (30.4)	49 (52.1)
Surgical resection and adjuvant CRT, *n* (%)	51 (39.8)	76 (56.7)	29 (63.0)	36 (38.3)
Surgical resection and other*, *n* (%)	<5 (N/A)^†^	<5 (N/A)^†^	<5 (N/A)^†^	9 (9.6)
Definitive non-surgical treatment, *N* (%)	125 (49.4)	255 (65.6)	84 (64.6)	144 (60.5)
CRT, *n* (%)	73 (58.4)	152 (59.6)	60 (71.4)	62 (43.1)
Radiotherapy, *n* (%)	40 (32.0)	72 (28.2)	15 (17.9)	72 (50.0)
Systemic treatment and radiotherapy, not started as CRT, *n* (%)	6 (4.8)	28 (11.0)	8 (9.5)	5 (3.5)
Other^‡^, *n* (%)	6 (4.8)	<5 (N/A)^†^	<5 (N/A)^†^	5 (3.5)

CRT, chemoradiotherapy; N/A, not applicable.

*“Other” included systemic treatment ± radiotherapy; ^†^Some groups could not be reported for data protection purposes (*n* < 5); therefore, % could not be reported; ^‡^“Other” included CRT and surgery, and radiotherapy and surgery.

**Figure 3:**
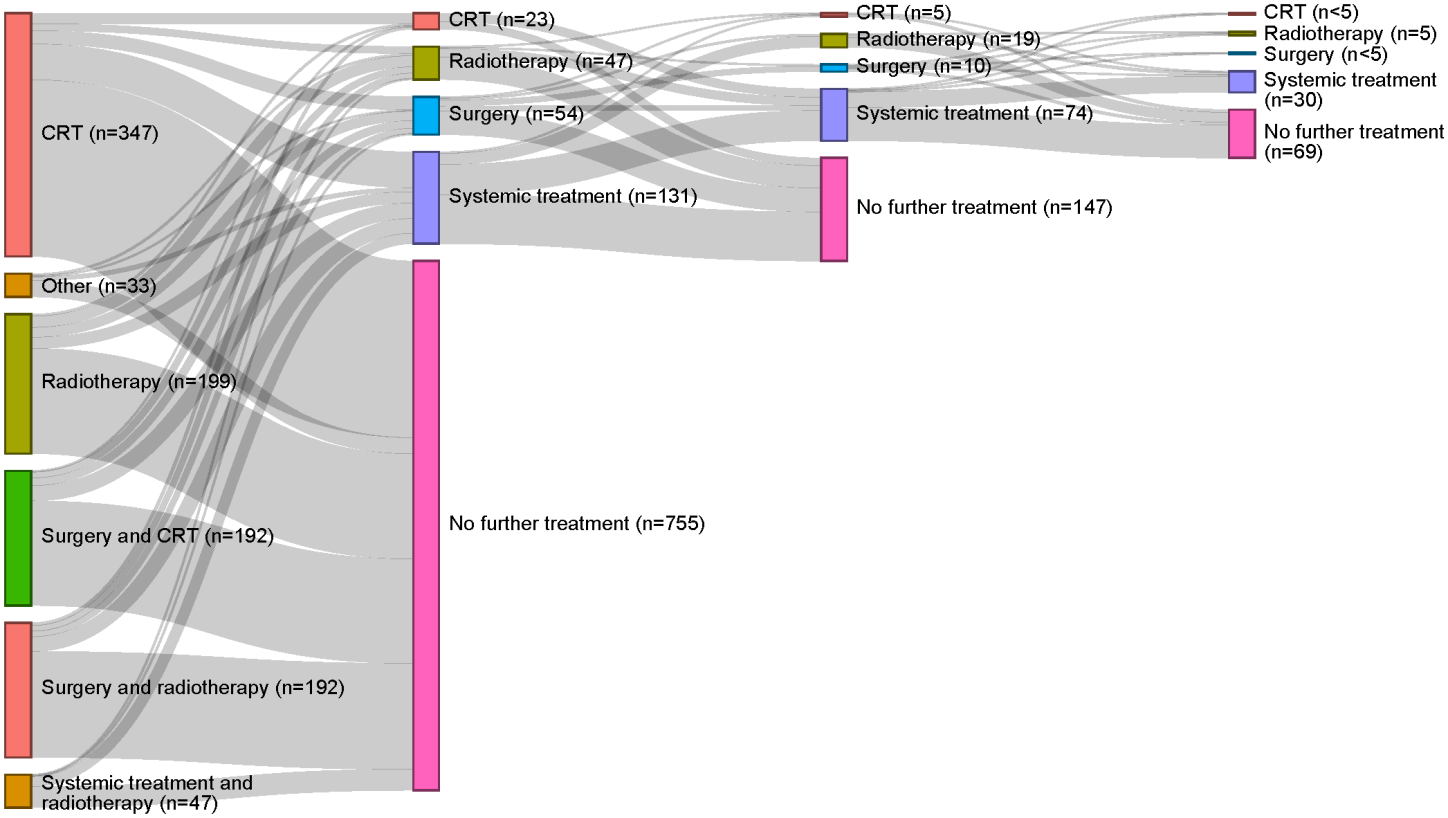
Treatment pathways identified for the overall study population. The first column reflects index treatment; each subsequent column represents a new treatment strategy. CRT, chemoradiotherapy.

**Figure 3** depicts the treatment pathways identified, providing the patient flow from index treatment through subsequent treatment strategies according to the treatment algorithm designed for this study. When applying this algorithm, 74.8% (755/1,010) of patients receiving index treatment for LA SCCHN received no subsequent SCCHN treatment. For those patients who did receive a subsequent treatment regimen, systemic treatments were mostly used (131/255). Of the 255 patients who received ≥1 subsequent treatment(s) after index treatment, most received only one additional treatment regimen, predominantly comprising chemo- and/or immunotherapy. Of 108 patients who received a second subsequent treatment regimen after index treatment, most received immunotherapy. See **Supplementary Tables S5, S6** for systemic treatments received subsequent to index treatment.

Approximately a fifth [21.6% (87/402)] of the patients who received surgical resection at index treatment received subsequent treatment. A slightly higher rate [27.6% (168/608)] of subsequent treatment was observed in the definitive non-surgical treatment cohort. See **Supplementary Figure S1** for treatment pathways by surgical resection and definitive non-surgical treatment cohorts.

### 3.4 Survival outcomes

**Figure 4:**
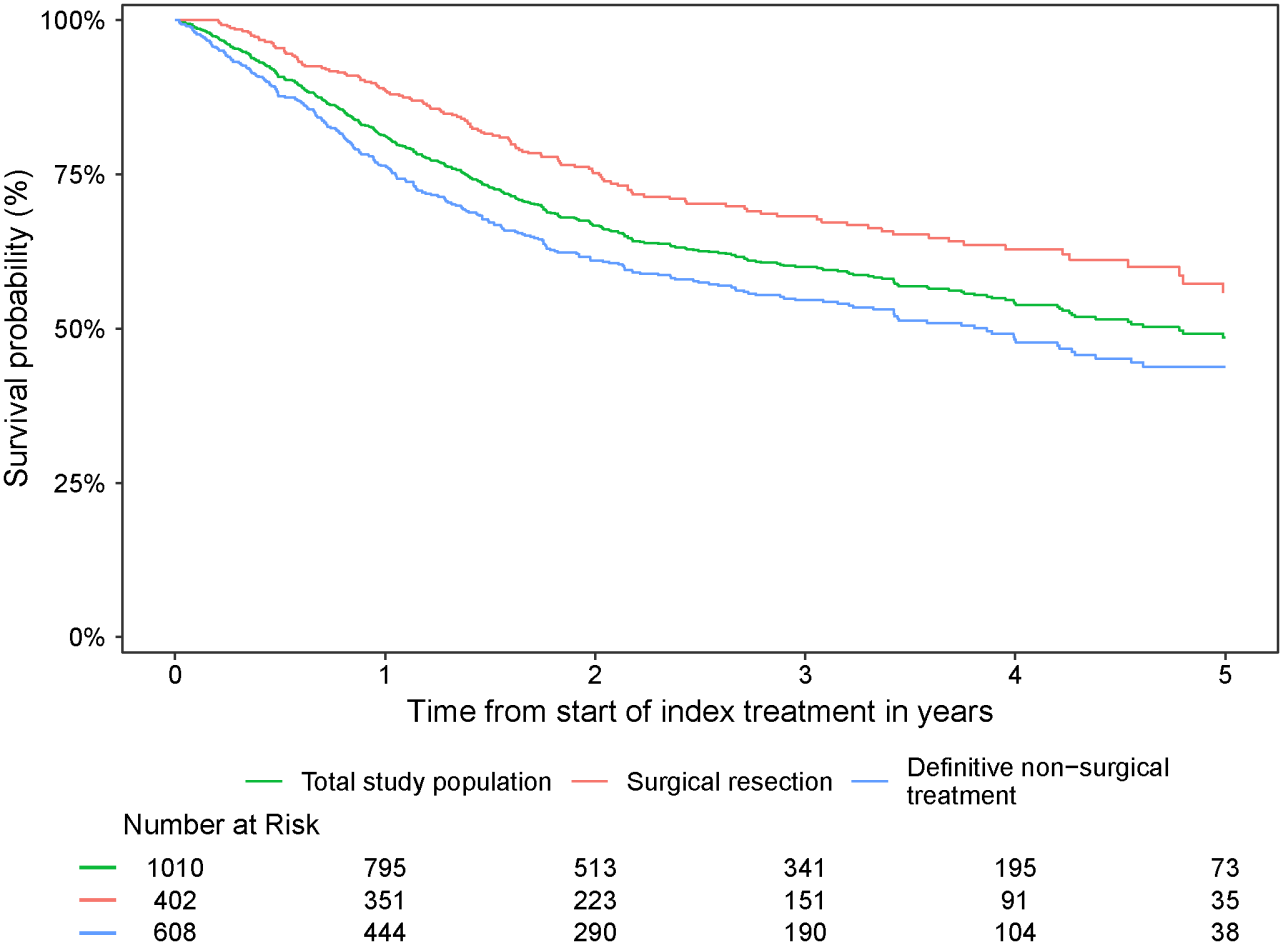
Survival probability from start of index treatment over time for the overall study population and by index treatment received.

Survival probabilities over time for the overall study population and by index treatment received are presented in **Figure 4**. The 5-year survival probability for the overall population was 48.5% (95% CI: 44.4%–53.1%) (**Table 4**). Surgical resection as index treatment was associated with 1-, 3-, and 5- year survival probabilities of 88.5% (95% CI:85.5%–91.7%), 68.2% (95% CI: 63.4%–73.4%), and 55.8% (95% CI:48.8%–63.7%), respectively. Definitive non-surgical treatment as index treatment was associated with 1-, 3-, and 5-year survival probabilities of 76.2% (95% CI:72.9%–79.7%), 54.6% (95% CI:50.5%–59.1%), and 43.8% (95% CI: 38.9%–49.4%), respectively.

**Table 4:** Survival probabilities at 1, 3, and 5 years for the overall study population and by primary tumor site.

Survival probability, % (95% CI)	Overall study population (*N* = 1,010)	Oral cavity (*N* = 253)	Oropharynx (*N* = 389)	Hypopharynx (*N* = 130)	Larynx (*N* = 238)
Surgical resection (*N* = 402)					
1 year	88.5 (85.4–91.7)	87.5 (81.9–93.4)	89.5 (84.4–94.8)	76.1 (64.7–89.5)	94.7 (90.3–99.3)
3 years	68.2 (63.4–73.4)	64.5 (56.0–74.4)	72.8 (65.0–81.6)	49.8 (35.9–69.2)	73.9 (64.8–84.4)
5 years	55.8 (48.8–63.7)	59.8 (49.9–71.5)	63.2 (53.2–75.0)	46.0 (32.0–66.2)	44.8 (28.6–70.4)
Definitive non-surgical treatment (*N* = 608)					
1 year	76.2 (72.9–79.7)	70.1 (62.5–78.7)	73.7 (68.5–79.3)	78.5 (70.2–87.8)	83.2 (77.3–89.6)
3 years	54.6 (50.5–59.1)	48.0 (39.5–58.5)	53.9 (47.8–60.9)	47.1 (36.4–60.9)	64.5 (56.5–73.6)
5 years	43.8 (38.9–49.4)	43.0 (34.1–54.3)	36.8 (28.0–48.4)	40.8 (29.5–56.5)	53.1 (43.6–64.6)

CI, confidence interval.

**Figure 5:**
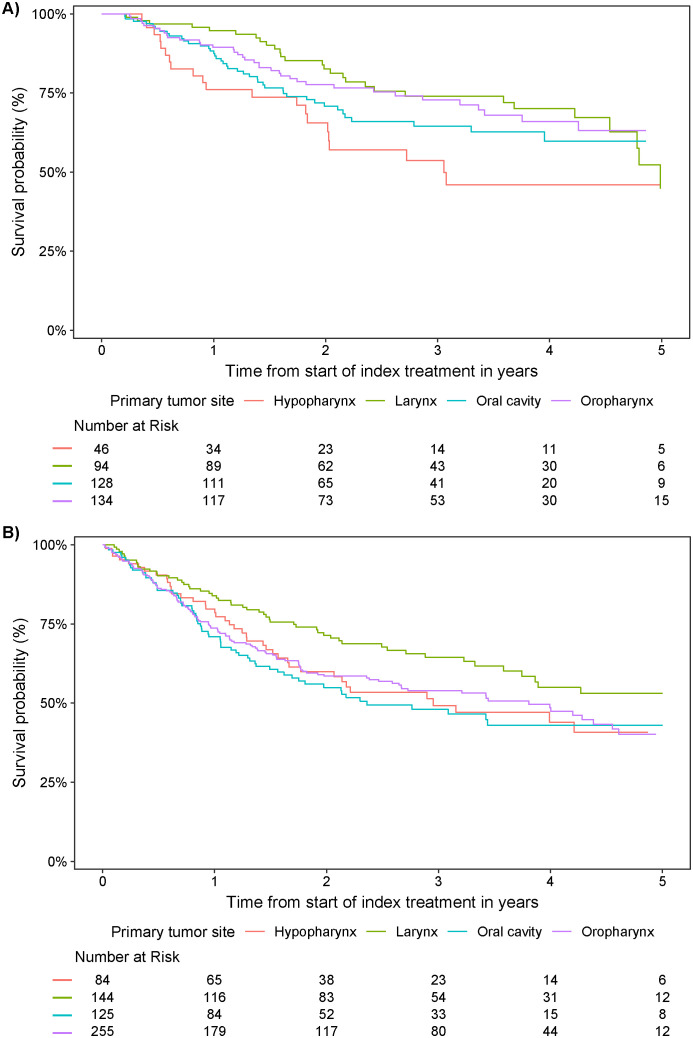
Survival probability from start of index treatment over time by primary tumor site in patients who received **(A)** surgical resection and **(B)** definitive non-surgical treatment.

Survival probabilities over time varied by primary tumor site and index treatment (**Figure 5**; **Table 4**). With the exception of laryngeal cancer, surgical resection resulted in a numerically higher survival probability at 5 years versus non-surgical treatment in all tumor types. Survival probabilities were consistently low at years 1, 3, and 5 for patients with hypopharyngeal cancer, regardless of index treatment regimen received. Patients with oral cavity cancer who did not receive surgical resection as part of index treatment also had low survival probabilities across years 1, 3, and 5.

Survival probabilities according to sex can be found in **Supplementary Figure S2**, but should be interpreted with caution given the low patient numbers in the female cohort at later time points.

## 4 Discussion

This study is the first to report real-world treatment patterns and associated survival outcomes for patients with LA SCCHN in Germany. The WIG2 database used for the analyses is representative of the whole SHI population and covers ~4.5 million persons insured by one of various German SHI providers (26). The WIG2 population has been validated with regard to age, sex, and morbidity (29). The incidence of LA SCCHN in the WIG2 population generally increased over time, consistent with reported epidemiology across the world (5).

Treatment of SCCHN remains a challenge for oncologists owing to heterogeneity in the patient population, stagnation in the treatment landscape, and plateauing survival rates (12, 25, 30, 31). Findings of this study demonstrate consistency with these issues in the German LA SCCHN population.

Prognosis and treatment depend on primary tumor site and stage at diagnosis (2, 8). Standard of care for patients with LA disease is widely accepted to be surgery plus adjuvant radiotherapy/CRT or, for patients not indicated for surgery, definitive CRT (2). Taking a holistic view of the results of this study, index treatments received largely adhere to these overarching recommendations.

Where feasible and appropriate, surgery is the foundation of treatment for LA SCCHN, with radical resection aiming to attain locoregional control (15–17, 30, 31). According to findings of this study, ~60% of patients with LA SCCHN did not undergo surgery. This rate is consistent with findings from other countries (32–34), but should be considered in the context of the exclusion of patients who received only surgery, so as not to include early-stage disease, which may have resulted in bias in the treatment split and underestimation of the true rate of resection. Though findings and comparisons should be cautiously approached due to the descriptive nature of this study, on average, patients who received non-surgical index treatment in this study were slightly older and had higher comorbidity indices than the cohort that underwent surgical resection after diagnosis; such characteristics are expected as they inherently render this group prognostically less favorable and less likely to be eligible for a surgical procedure. Divergence in cohort characteristics precludes a fair comparison of outcomes related to specific interventions.

For those patients who cannot or choose not to receive surgery, definitive CRT has been the mainstay of LA SCCHN treatment for over three decades (19); the prognosis for these patients is reportedly worse than for those who receive surgery (35), likely due to unfavorable patient and disease characteristics. This study numerically supports these findings.

In line with European and German guidelines, patients for whom identifiable systemic treatment formed part of index treatment predominantly received cisplatin (2, 15–17). Cisplatin has been used to treat cancer since the 1970s, but is associated with known toxicities (36–38). Factors that render patients ineligible include poor performance status, advanced age, poor renal function, or hearing loss (37). However, the identification of generic chemotherapy treatments was limited to patients in the outpatient setting and it is difficult to draw more detailed conclusions on guideline adherence with respect to specific systemic treatments. The relatively large proportion of patients in both cohorts being treated with definitive radiotherapy might be explained by their characteristics, i.e., age or comorbidities precluding systemic treatment. These rates are in line with those seen in other geographies, including the UK and US (33, 39). Owing to limitations in the capture of these data within claims records and the heterogeneity of these patients, extreme caution is emphasized when interpreting or comparing systemic treatments.

Despite the curative intent of treatment for LA SCCHN (14), over half the population of this large dataset did not survive beyond 5 years. Locoregional recurrences are reported to occur in 30%–40% of patients with advanced SCCHN and are difficult to manage (30). The finding of this study that only a quarter of patients go on to receive subsequent treatment regimens after index treatment appear to substantiate this, but it is not possible to definitively conclude the reasons behind it; it could be a product of the treatment algorithm applied, but could equally be reflective of the features of the patient population at this stage in their disease, precluding or preferentially not receiving further treatment. This finding is, however, consistent with rates reported elsewhere (12, 40).

The LA SCCHN population, with divergent risk factors and primary sites, is known to be heterogeneous, as reflected in this study. This contributed to the variation in treatment approaches and outcomes by primary tumor location observed. Findings were generally expected; with respect to index treatment received, patients with oral cavity cancer had similar rates of definitive non-surgical treatment and surgical resection. All other tumor sites were treated more frequently by definitive non-surgical treatment. Despite the functional challenges of resecting within the oral cavity, it is often easier to achieve larger margins than at other locations, which may explain this finding (30).

Given that patients eligible for surgery have a more favorable prognosis, it is not surprising that survival probabilities are higher in patients with tumor resection as index treatment. This is reflected across patients with LA SCCHN, independent of the exact localization, but most significantly in patients with oropharyngeal cancer. This tumor type is commonly linked to HPV positivity, which is acknowledged to be associated with a better prognosis than other SCCHN types (2, 8, 41). For tumors of the hypopharynx, survival probabilities were improved, but not dramatically, for patients receiving surgery versus definitive non-surgical treatment. Interestingly, in patients with laryngeal tumors, survival probabilities at later follow-up time points (i.e., from year 5 onwards) were numerically higher with definitive non-surgical treatments than those who received surgery; despite this being contradictory to some published studies (42, 43), a possible hypothesis for this is the frequent implementation of a laryngeal preservation approach in Germany, whereby patients receive induction therapy, followed by definitive radiotherapy or CRT (rather than surgery) in those who have a very good response (2, 44, 45). Data in this area are somewhat inconclusive (46, 47), and results herein should be interpreted within the context of the sample size and censoring constraints.

This study reported a ratio of roughly 4:1 male:female patients, which is in line with other German observational studies (6, 48). There was some variation in demographics and tumor site by sex; nonetheless, choice of index treatment was similar between male and female patients. Survival probabilities to year 5 were slightly higher for female patients at all time points. Information for female patients in this database could inform interesting future analyses, given the relative lack of research in this cohort.

### 4.1 Limitations

Certain types of bias, such as selection and information bias, are inherent to all observational studies. Studies that utilize a database as the source of information are limited by the fields captured and rely on accurate reporting by users. Insurance claims database studies can be particularly challenging as data are collected for the purpose of payment and not research. The use and limitations of German claims data have been described in previous publications (49, 50).

A challenge of using insurance claims data for the clinical questions asked in this study was the lack of capture of certain parameters, notably tumor stage, histology, HPV status, and performance status. Because of a lack of tumor stage and histology data, the design of sophisticated inclusion and exclusion criteria to select the population of interest was required. Based on clinical guidelines and expert advice, data for treatments and sequences thereof were used to identify patients with LA SCCHN, which could result in potential misclassification. It is unclear whether this would under- or overrepresent the true LA SCCHN population in Germany or lead to an underestimation of surgical resection rates.

Claims data do not capture explicit treatment lines or clinical information on cancer progression or recurrence. For the purposes of this study, a treatment algorithm, with various assumptions, was designed to identify the sequence of treatments received according to data available. As a result, it is not possible to definitively discern whether treatment is an actual subsequent regimen or whether it was indicated for metastatic or recurrent cancer. Findings for subsequent treatment regimens beyond index treatment are further constrained by small sample sizes and should be considered in this context.

While the database captures daily information for prescriptions and hospital visits, certain pieces of information (such as diagnoses made by practitioners in the outpatient setting or primary care) are only available on a quarterly basis due to quarterly billing in the outpatient setting. Individual treatments administered are also difficult to comprehensively describe since, with the exception of certain high-cost drugs, treatment costs (e.g., cisplatin or carboplatin) normally form part of a lump sum and are not individually reported for inpatient stays. Furthermore, chemotherapy is also often captured via an unspecific chemotherapy code as opposed to the exact regimen used. Switches in treatment that may have occurred during a hospital stay may also not be discernible, and may only be observable through the outpatient prescriptions following the inpatient stay. This issue may be further compounded by the fact that a claim for a filled prescription is not an indication that the medication was taken as prescribed.

The finding that the majority of patients received definitive non-surgical treatment should be considered in the context of the patient demographics and the exclusion of patients who received surgery alone so as not to include early-stage disease. This may have resulted in bias in the treatment split and underestimation of the true rate of resection.

The database does not contain information on HPV status, so assessment of treatment patterns and outcomes by HPV positivity was not possible. The importance of HPV positivity has only recently gained importance, leading to the 2019 TNM-8 status change that has led to challenges in classification in clinical practice, which may have impacted inclusion and conclusions in the present study (9–11). HPV status is considered to be a therapy-independent prognostic factor.

It is acknowledged that risk factors (tobacco smoking and alcohol use) and comorbidities are likely underreported due to the claims-based nature of the database used. The presence of smoking and alcohol use itself does not impact treatment choice, and their reporting is not mandated when submitting claims. Furthermore, the increasing prevalence of HPV-related SCCHN could provide a potential reason for lower-than-expected rates of tobacco smoking and alcohol abuse; however, this claim cannot be validated.

### 4.2 Conclusions

This real-world observational study of a large German dataset provides valuable clinical insight into the treatment patterns and associated survival outcomes for patients with LA SCCHN in Germany. Though treatment choice upon diagnosis was largely aligned to guideline recommendations, most patients received no subsequent SCCHN treatment and almost half had died within 5 years. The heterogeneous LA SCCHN population contributed to varied findings in treatment approaches and outcomes. Findings of this study underscore the unmet need and amplify the call for superior treatment options across all patients with LA SCCHN.

## Data Availability

The data analyzed in this study is subject to the following licenses/restrictions: The data sets generated and analyzed as part of this study are not publicly accessible for reasons of data protection. Requests to access these datasets should be directed to Nancy Schoenherr, nancy.schoenherr@merckgroup.com.
