# Supplementary material for: Real-world treatment patterns and survival outcomes in patients with locally advanced squamous cell carcinoma of the head and neck in Germany using claims data
Source: Front Oncol. 2025 Jun 5;15:1547311. doi: 10.3389/fonc.2025.1547311 (PMC12177216; doi:10.3389/fonc.2025.1547311)
Supplement: Supplementary file 1 [file DataSheet1.docx]

Supplementary material

**Contents**

[1. Inclusion and exclusion criteria 2](#_Toc184115906)

[2. Incidence of LA SCCHN 6](#_Toc184115907)

[3. Baseline demographics and index treatment by sex 7](#_Toc184115908)

[4. Systemic treatment as part of regimens received post index-treatment 8](#_Toc184115909)

[5. Treatment pathways 9](#_Toc184115910)

[6. Survival outcomes 10](#_Toc184115911)

**List of Figures**

[Figure S1. Treatment pathways identified for patients that received (A) surgical resection (N=402) and (B) definitive non-surgical treatment (N=608) as part of index treatment 9](#_Toc184115912)

[Figure S2. Survival probability from start of index treatment over time by sex 10](#_Toc184115913)

**List of Tables**

[Table S1. ICD-10 GM codes used to identify patients with head and neck cancer 2](#_Toc184115914)

[Table S2. Codes/definitions used to identify SCCHN treatments 4](#_Toc184115915)

[Table S3. Incidence of LA SCCHN in Germany from 2016 to 2020 and extrapolated to the SHI population 6](#_Toc184115916)

[Table S4. Baseline characteristics and index treatment by sex 7](#_Toc184115917)

[Table S5. Systemic treatment regimens received as the first subsequent treatment strategy post-index treatment in patients for whom systemic treatment was identifiable 8](#_Toc184115918)

[Table S6. Systemic treatments received as the second subsequent treatment strategy post-index treatment in patients for whom systemic treatment was identifiable 8](#_Toc184115919)

# Inclusion and exclusion criteria

## Inclusion criteria

Individuals were included in the study only if they met the following criteria:

1. Patients with an initial diagnosis of head and neck cancer between 1 January 2016 and 31 December 2020, defined as:
   1. at least one International Classification of Diseases 10th edition German Modification (ICD-10-GM) (1) diagnostic code of head and neck cancer (Table S1) as inpatient diagnosis between 1 January 2016 and 31 December 2020; or
   2. at least two confirmed outpatient diagnoses of head and neck cancer within 365 days, between 1 January 2016 and 31 December 2020.
2. Patients aged ≥18 years at the first head and neck cancer diagnosis (index date).
3. Patients with at least 24 months of continuous enrolment prior to index date (to ensure at least 24 months without prior diagnosis of head and neck cancer) or death.

## Exclusion criteria

Individuals were excluded from the study if they met any of the following criteria:

1. At least one ICD-10-GM code (2) for metastatic cancer or cancer of unknown primary (CUP): C77 (except C77.0), C78, C79, C80 up to 8 weeks after the index diagnosis.
2. Any primary cancer other than head and neck, with the exception of malignant neoplasms of the skin (ICD-10-GM C00–C75 except C44 and the codes listed in Table S1) prior to the index date or within three months of the index date since a substantial proportion of these are squamous cell cancers that occur on the head or neck (2).
3. No treatment for SCCHN in the quarter of the first diagnosis of LA SCCHN or the subsequent quarter.
4. Treatment(s) indicating metastatic disease (Table S2).
5. Surgery only as index treatment (patients receiving surgery in combination with other treatments were not excluded).
6. Systemic therapy only (either as monotherapy or in combination with other systemic therapies) as index treatment following diagnosis, with the exception of patients with a larynx carcinoma [C32] treated with paclitaxel/docetaxel + cisplatin/carboplatin + fluorouracil [TFP regimen] according to guideline recommendations (3-7).
7. Participation in a clinical trial or evidence of clinical trial enrolment (ICD-10-GM Z00.6) after the index date.
8. Ambiguous tumor site diagnoses.

Table S1. ICD-10 GM codes used to identify patients with head and neck cancer

| **Site/cancer type** | **ICD-10 code** | **Diagnosis** |
| --- | --- | --- |
| Oral cavity | | |
| Malignant neoplasm of lip | C00.3 | Upper lip, inner aspect |
|  | C00.4 | Lower lip, inner aspect |
|  | C00.5 | Lip, unspecified, inner aspect |
| Malignant neoplasm of other and unspecified parts of tongue | C02.0 | Dorsal surface of tongue |
|  | C02.1 | Border of tongue |
|  | C02.2 | Ventral surface of tongue |
|  | C02.3 | Anterior two-thirds of tongue, part unspecified |
|  | C02.4 | Lingual tonsil |
|  | C02.8 | Overlapping lesion of tongue |
|  | C02.9 | Tongue, unspecified |
| Malignant neoplasm of gum | C03.0 | Upper gum |
|  | C03.1 | Lower gum |
|  | C03.9 | Gum, unspecified |
| Malignant neoplasm of floor of mouth | C04.0 | Anterior floor of mouth |
|  | C04.1 | Lateral floor of mouth |
|  | C04.8 | Overlapping lesion of floor of mouth |
|  | C04.9 | Floor of mouth, unspecified |
| Malignant neoplasm of palate | C05.0 | Hard palate |
|  | C05.1 | Soft palate |
|  | C05.2 | Uvula |
|  | C05.8 | Overlapping lesion of palate |
|  | C05.9 | Palate, unspecified |
| Malignant neoplasm of other and unspecified parts of mouth | C06.0 | Cheek mucosa |
|  | C06.1 | Vestibule of mouth |
|  | C06.2 | Retromolar area |
|  | C06.8 | Overlapping lesion of other and unspecified parts of mouth |
| Oropharynx | | |
| Malignant neoplasm of base of tongue | C01 | Malignant neoplasm of base of tongue |
| Malignant neoplasm of tonsil | C09.0 | Tonsillar fossa |
|  | C09.1 | Tonsillar pillar (anterior)(posterior) |
|  | C09.8 | Overlapping lesion of tonsil |
|  | C09.9 | Tonsil, unspecified |
| Malignant neoplasm of oropharynx | C10.0 | Vallecula |
|  | C10.1 | Anterior surface of epiglottis |
|  | C10.2 | Lateral wall of oropharynx |
|  | C10.3 | Posterior wall of oropharynx |
|  | C10.4 | Branchial cleft |
|  | C10.8 | Overlapping lesion of oropharynx |
|  | C10.9 | Oropharynx, unspecified |
| Malignant neoplasm of other and ill-defined sites in the lip, oral cavity and pharynx | C14.2 | Waldeyer ring |
| Hypopharynx |  |  |
| Malignant neoplasm of hypopharynx | C12 | Malignant neoplasm of piriform sinus |
|  | C13.0 | Post cricoid region |
|  | C13.1 | Aryepiglottic fold, hypopharyngeal aspect |
|  | C13.2 | Posterior wall of hypopharynx |
|  | C13.8 | Overlapping lesion of hypopharynx |
|  | C13.9 | Overlapping lesion of hypopharynx |
| Larynx | | |
| Malignant neoplasm of larynx | C32.0 | Glottis |
|  | C32.1 | Supraglottis |
|  | C32.2 | Subglottis |
|  | C32.3 | Laryngeal cartilage |
|  | C32.8 | Overlapping lesion of larynx |
|  | C32.9 | Larynx, unspecified |

Table S2. Codes/definitions used to identify SCCHN treatments

| **Variable** | **Code/Definition** |
| --- | --- |
| **Systemic therapy** | |
| Bleomycin | ATC: L01DC01 |
| Cisplatin | ATC L01XA01 |
| Carboplatin | ATC L01XA02 |
| Fluorouracil | ATC L01BC02 |
| Cetuximab | ATC L01FE01, OPS 6-001.a |
| Paclitaxel | ATC L01CD01, OPS 6-001.f |
| Docetaxel | ATC L01CD02, OPS 6-002.h |
| Mitomycin C | ATC L01DC03 |
| Methotrexate | ATC L01BA01, ATC L04AX03 |
| Nivolumab | ATC L01XC17, ATC L01FF01, OPS 6-008.m |
| Pembrolizumab | ATC L01XC18, ATC L01FF02, OPS 6-009.3 |
| Chemotherapy in hospital | OPS 8-542, 8-543, 8-544 |
| **Definitive surgery** | |
| Radical surgery for oropharynx | OPS 5-290 Pharyngotomy |
| Radical surgery for hypopharynx | OPS 5-295 Partial resection of pharynx [pharyngectomy]  OPS 5-296 Radical resection of the pharynx [pharyngectomy] |
| Radical surgery for larynx | OPS 5-301 Hemilaryngectomy  OPS 5-302 Other partial laryngectomy  OPS 5-303 Laryngectomy |
| Radical surgery for oral cavity | OPS 5-251 Partial glossectomy  OPS 5-252 Glossectomy  OPS 5-277 Resection of the floor of the mouth with plastic reconstruction  OPS 5-278 Resection of the cheek with plastic reconstruction  OPS 5-282 Tonsillectomy with adenotomy |
| Neck dissection in combination with any of the surgical codes above | OPS 5-403 Radical cervical lymphadenectomy  OPS 5-402.0 Regional lymphadenectomy  Only, if used in combination with any of the surgical codes above |
| **Radiotherapy** | |
| Radiotherapy | EBM 25320 Irradiation with telecobalt device (benign/malignant) or linear accelerator (benign)  EBM 25321 Irradiation with linear accelerator for malignant diseases  OPS 8-522 High-voltage radiotherapy  OPS 8-523 Other high-voltage radiation therapy  OPS 8-522.9 Intensity modulated RT (IMRT) |

**References for section**

1. Bundesinstitut für Arzneimittel und Medizinprodukte. ICD-10-GM Version 2020. Systematisches Verzeichnis, Internationale statistische Klassifikation der Krankheiten und verwandter Gesundheitsprobleme, 10. Revision, Stand: 20. September 2019. im Auftrag des Bundesministeriums für Gesundheit (BMG) unter Beteiligung der Arbeitsgruppe ICD des Kuratoriums für Fragen der Klassifikation im Gesundheitswesen (KKG). 2019.

2. Das Robert Koch-Institut ist ein Bundesinstitut im Geschäftsbereich des Bundesministeriums für Gesundheit. Cancer in Germany 2019/2020. 2024 [Available from: <https://www.krebsdaten.de/Krebs/EN/Content/Publications/Cancer_in_Germany/cancer_in_germany_node.html>].

3. Bootz F. S3-Leitlinie Diagnostik, Therapie und Nachsorge des Larynxkarzinoms. Der Radiologe. 2020;60(11):1052-7.

4. Leitlinienprogramm Onkologie. Diagnostik, Therapie und Nachsorge des Larynxkarzinoms, Langversion 1.1. AWMF-Registernummer: 017/076OL 2019 [Available from: <https://www.leitlinienprogramm-onkolo-gie.de/fileadmin/user_upload/Downloads/Leitlinien/Larynxkarzinom/Version1.1/LL_Larynxkarzinom_Langversion_1.1.pdf>].

5. Leitlinienprogramm Onkologie. S3-Leitlinie Diagnostik und Therapie des Mundhöhlenkarzinoms, Langversion 3.0. AWMF Registernummer: 007/100OL 2021 [Available from: <https://www.leitlinienprogramm-onkolo-gie.de/fileadmin/user_upload/Downloads/Leitlinien/Mundhoehlenkarzinom/Version_3/LL_Mundhoehlenkarzinom_Langversion_3.0.pdf>].

6. Leitlinienprogramm Onkologie. S3-Leitlinie Diagnostik, Therapie, Prävention und Nachsorge des Oro- und Hypopha-rynxkarzinoms. WMF-Registernummer: 017-082OL 2024 [Available from: <https://www.leitlinienprogramm-onkologie.de/fileadmin/user_upload/Downloads/Leitlinien/Oro-_und_Hypopharynxkarzinom/LL_Oro-_und_Hypopharynxkarzinom_Langversion_1.0.pdf>].

7. Machiels JP, René Leemans C, Golusinski W, Grau C, Licitra L, Gregoire V. Squamous cell carcinoma of the oral cavity, larynx, oropharynx and hypopharynx: EHNS-ESMO-ESTRO Clinical Practice Guidelines for diagnosis, treatment and follow-up. Ann Oncol. 2020;31(11):1462–75.

# Incidence of LA SCCHN

Table S3. Incidence of LA SCCHN in Germany from 2016 to 2020 and extrapolated to the SHI population

| **Year** | **WIG2 database (N=1,010)** | | | **SHI (extrapolated)** | |
| --- | --- | --- | --- | --- | --- |
|  | **n** | **Incidence per 100,000 adults** | **n** | | **Incidence per 100,000 adults** |
| **2016** | 182 | 7.10 | 3,963 | | 6.66 |
| **2017** | 210 | 8.27 | 4,626 | | 7.70 |
| **2018** | 226 | 9.04 | 5,049 | | 8.35 |
| **2019** | 183 | 7.41 | 4,072 | | 6.72 |
| **2020** | 209 | 8.48 | 4,680 | | 7.70 |

LA, locally advanced; SCCHN, squamous cell carcinoma of the head and neck; SHI, statutory health insurance; WIG2, Wissenschaftliches Institut für Gesundheitsökonomie und Gesundheitssystemforschung.

# Baseline demographics and index treatment by sex

Table S4. Baseline characteristics and index treatment by sex

|  | **Female (N=183)** | **Male (N=827)** |
| --- | --- | --- |
| **Age (years), mean (SD)** | 63.1 (9.95) | 62.4 (9.36) |
| **Age categories, n (%)** | | |
| **≤65** | 112 (61.2) | 535 (64.7) |
| **>65** | 71 (38.8) | 292 (35.3) |
| **≤70** | 139 (76.0) | 661 (79.9) |
| **>70** | 44 (24.0) | 166 (20.1) |
| **Primary tumor site, n (%)** | | |
| **Oropharynx** | 79 (43.2) | 310 (37.5) |
| **Oral cavity** | 57 (31.1) | 196 (23.7) |
| **Larynx** | 29 (15.8) | 209 (25.3) |
| **Hypopharynx** | 18 (9.8) | 112 (13.5) |
| **Comorbidity indices, mean (SD)** | | |
| **ECI** | 4.1 (6.98) | 6.7 (8.59) |
| **CCI** | 2.1 (2.84) | 2.3 (2.75) |
| **Risk factors, n (%)** | | |
| **Tobacco consumption** | 49 (26.8) | 254 (30.7) |
| **Alcohol abuse** | 26 (14.2) | 237 (28.7) |
| **Index treatment, n (%)** | | |
| **Surgical resection** | 75 (41.0) | 342 (41.4) |
| **Definitive non-surgical treatment** | 108 (59.0) | 485 (58.6) |

CCI, Charlson Comorbidity Index; ECI, Elixhauser Comorbidity Index; SD, standard deviation.

# Systemic treatment as part of regimens received post index-treatment

Table S5. Systemic treatment regimens received as the first subsequent treatment strategy post-index treatment in patients for whom systemic treatment was identifiable

| **Treatment(s)** | **Patients with identifiable regimen (N=125)** |
| --- | --- |
| Cetuximab combinations*, n (%) | 47 (37.6) |
| Immunotherapy monotherapy, n (%) | 35 (28.0) |
| Chemotherapy monotherapy and combinations^†^, n (%) | 17 (13.6) |
| Immunotherapy in combination with chemotherapy, n (%) | 14 (11.2) |
| Cetuximab monotherapy, n (%) | 6 (4.8) |
| Other^‡^, n (%) | 6 (4.8) |

*Includes platinum + fluorouracil + cetuximab (EXTREME), docetaxel + cisplatin + cetuximab (TPEx) and other cetuximab combinations with chemotherapies; ^†^Includes platinum-based treatments, taxanes or fluorouracil (5FU) as monotherapies or in combination with each other; ^‡^‘Other’ included TPEx + immunotherapy, cetuximab +immunotherapy, EXTREME + immunotherapy.

Table S6. Systemic treatments received as the second subsequent treatment strategy post-index treatment in patients for whom systemic treatment was identifiable

| **Treatment(s)** | **Patients with identifiable regimen (N=70)** |
| --- | --- |
| Immunotherapy monotherapy, n (%) | 36 (51.4) |
| Chemotherapy monotherapy and combinations*, n (%) | 14 (20.0) |
| Cetuximab combinations^†^, n (%) | 10 (14.3) |
| Cetuximab monotherapy, n (%) | 5 (7.1) |
| Immunotherapy in combination with chemotherapy, n (%) | 5 (7.1) |

*Includes platinum-based treatments, taxanes or 5FU as monotherapies or in combination; ^†^Includes EXTREME, TPEx and other cetuximab-containing chemotherapy combinations.

# Treatment pathways

**(A)
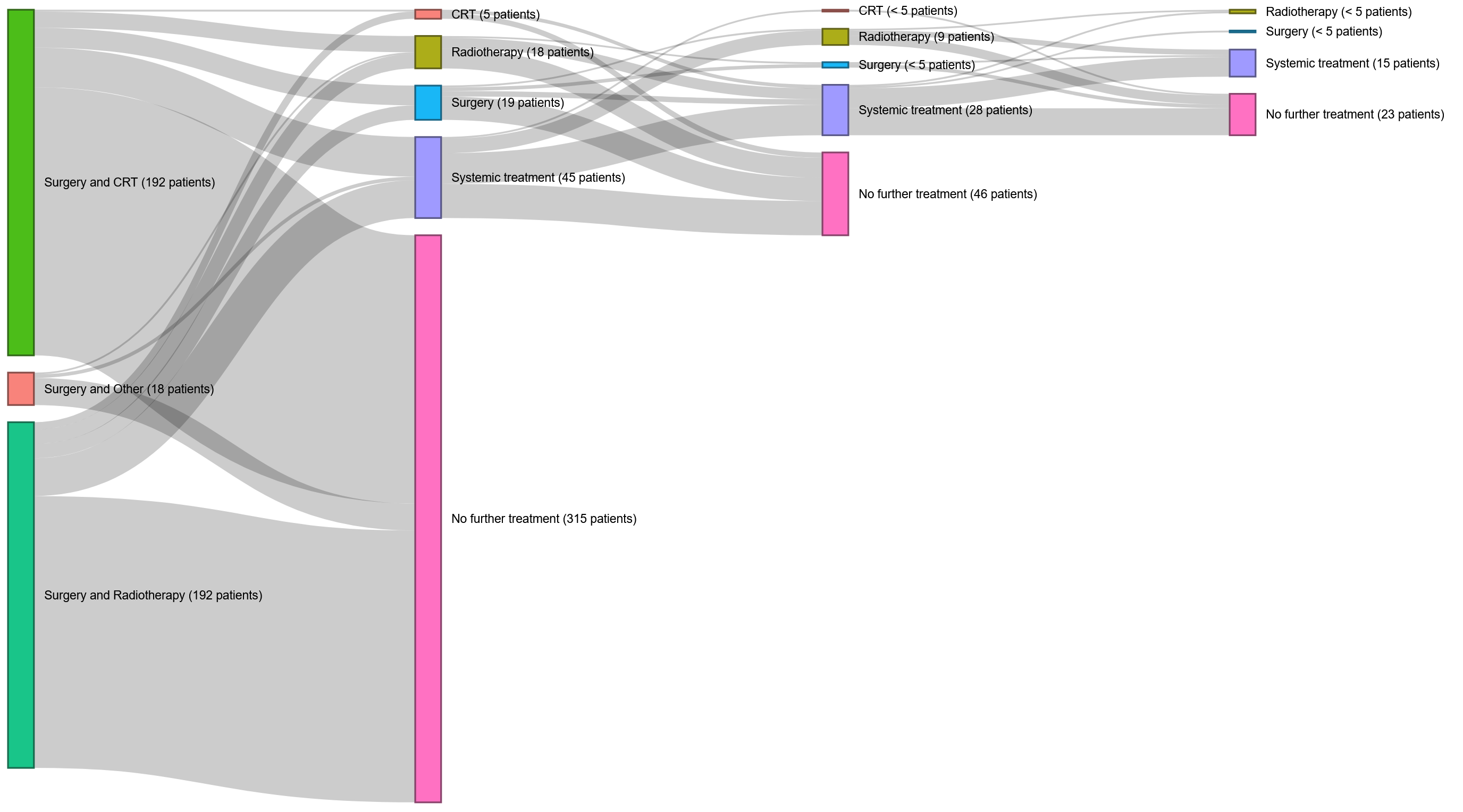
**

**(B)
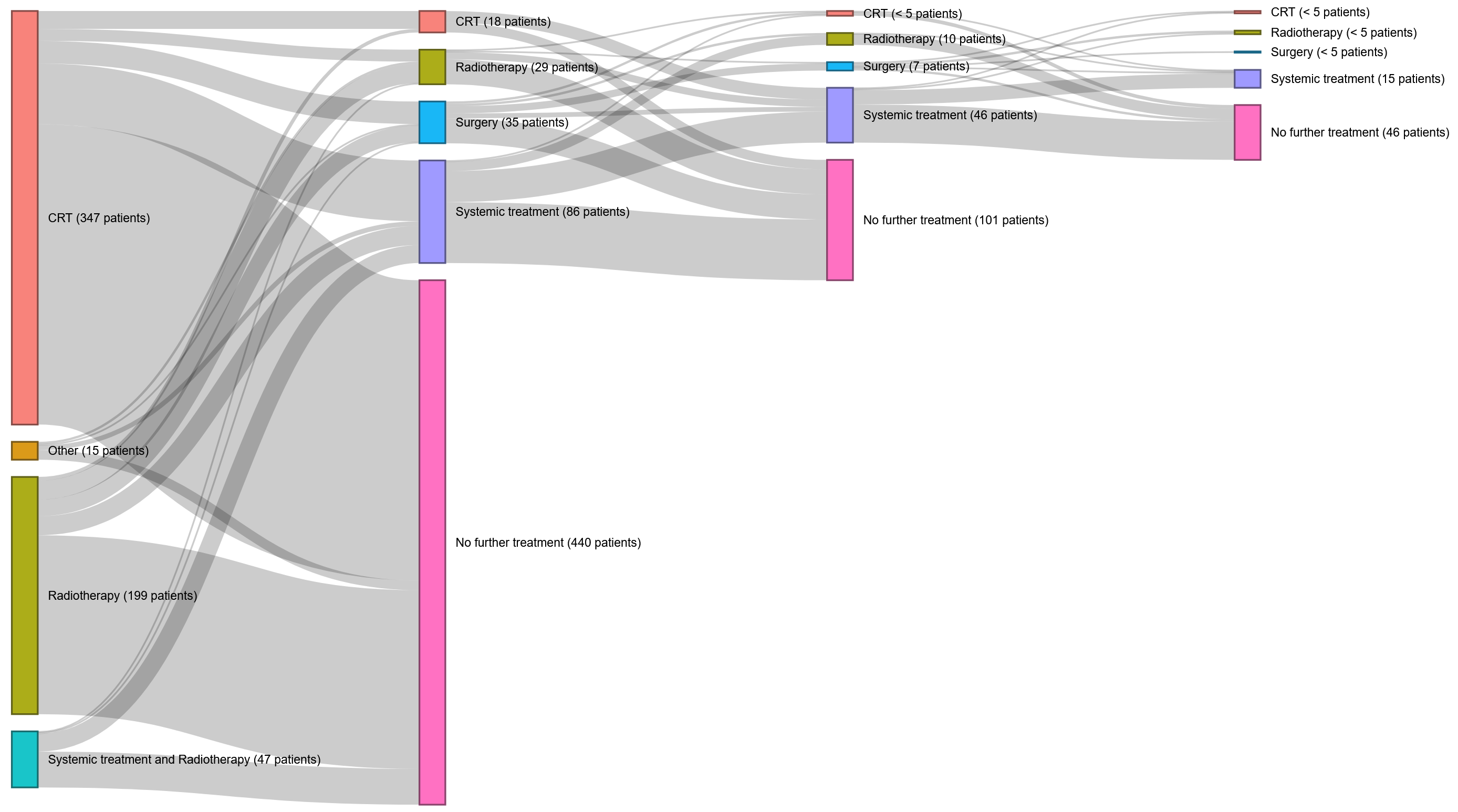
**

Figure S1. Treatment pathways identified for patients that received (A) surgical resection (N=402) and (B) definitive non-surgical treatment (N=608) as part of index treatment

CRT, chemoradiotherapy.

The first column reflects index treatment received; each subsequent column represents a new treatment strategy.

# Survival outcomes


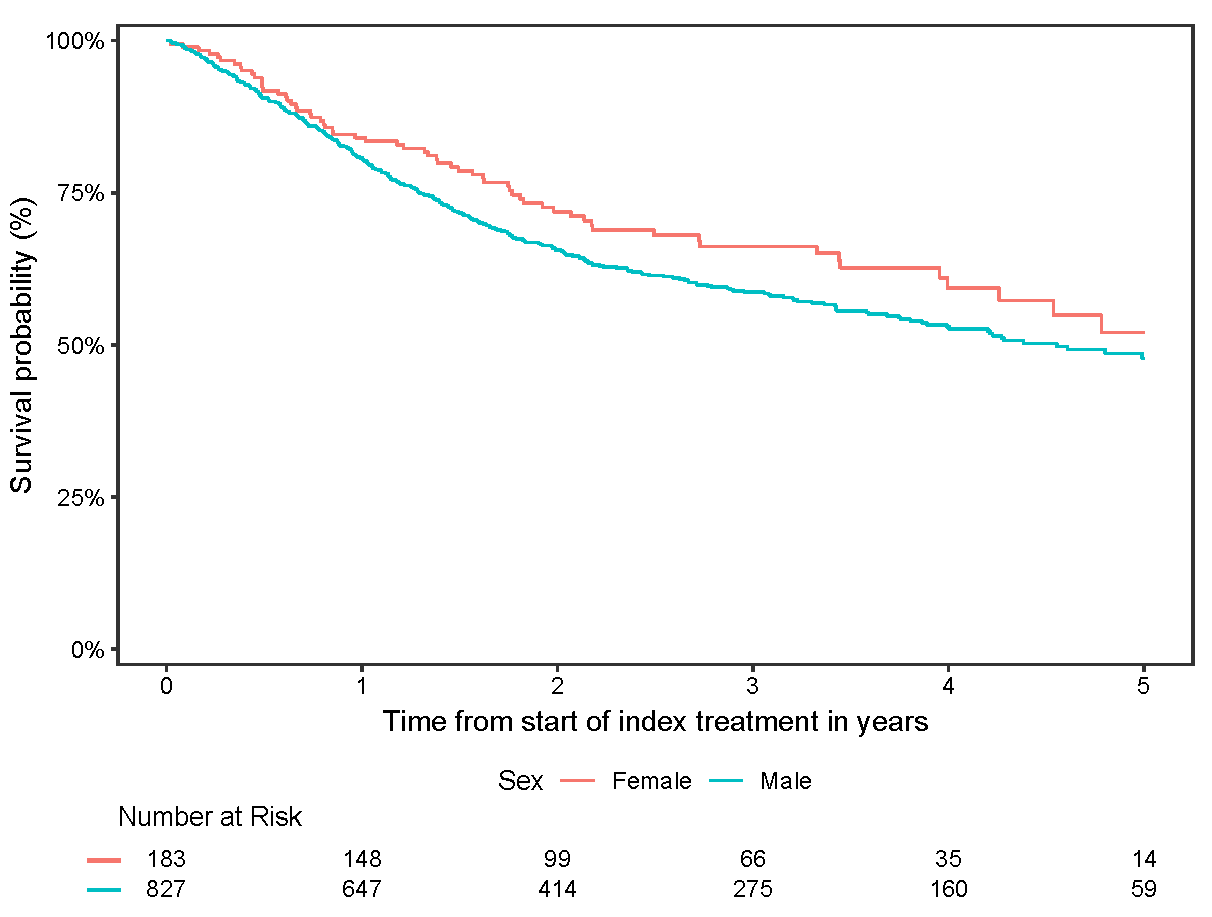


Figure S2. Survival probability from start of index treatment over time by sex
